# Polydendrocytes Display Large Lineage Plasticity following Focal Cerebral Ischemia

**DOI:** 10.1371/journal.pone.0036816

**Published:** 2012-05-10

**Authors:** Pavel Honsa, Helena Pivonkova, David Dzamba, Marcela Filipova, Miroslava Anderova

**Affiliations:** 1 Department of Cellular Neurophysiology, Institute of Experimental Medicine, Academy of Sciences of the Czech Republic, Prague, Czech Republic; 2 2nd Faculty of Medicine, Charles University, Prague, Czech Republic; University of Iowa, United States of America

## Abstract

Polydendrocytes (also known as NG2 glial cells) constitute a fourth major glial cell type in the adult mammalian central nervous system (CNS) that is distinct from other cell types. Although much evidence suggests that these cells are multipotent *in vitro*, their differentiation potential *in vivo* under physiological or pathophysiological conditions is still controversial.

To follow the fate of polydendrocytes after CNS pathology, permanent middle cerebral artery occlusion (MCAo), a commonly used model of focal cerebral ischemia, was carried out on adult NG2creBAC:ZEG double transgenic mice, in which enhanced green fluorescent protein (EGFP) is expressed in polydendrocytes and their progeny. The phenotype of the EGFP^+^ cells was analyzed using immunohistochemistry and the patch-clamp technique 3, 7 and 14 days after MCAo. In sham-operated mice (control), EGFP^+^ cells in the cortex expressed protein markers and displayed electrophysiological properties of polydendrocytes and oligodendrocytes. We did not detect any co-labeling of EGFP with neuronal, microglial or astroglial markers in this region, thus proving polydendrocyte unipotent differentiation potential under physiological conditions. Three days after MCAo the number of EGFP^+^ cells in the gliotic tissue dramatically increased when compared to control animals, and these cells displayed properties of proliferating cells. However, in later phases after MCAo a large subpopulation of EGFP^+^ cells expressed protein markers and electrophysiological properties of astrocytes that contribute to the formation of glial scar. Importantly, some EGFP^+^ cells displayed membrane properties typical for neural precursor cells, and moreover these cells expressed doublecortin (DCX) – a marker of newly-derived neuronal cells. Taken together, our data indicate that polydendrocytes in the dorsal cortex display multipotent differentiation potential after focal ischemia.

## Introduction

In general, the adult central nervous system (CNS) possesses a limited capacity for regeneration after injury, including ischemia. Following ischemic injury, neural tissue recovery is accompanied by the formation of reactive astrogliosis; this process is vital for isolating necrotic tissue from its uninjured surroundings, but concurrently, it markedly impedes regenerative processes. Shortly after ischemia, a series of ionic, neurotransmitter and oxidative radical imbalances occurs that lead to the activation of microglia and subsequently to an increased number of reactive astrocytes. Both cell types release cytokines and other soluble products [1] that play an important role in consecutive processes, including the apoptosis of oligodendrocytes [2] and neurons [3]. Besides the main, well characterized cell types, other cells including polydendrocytes, endothelial cells and pericytes exist in neural tissue; however, our knowledge regarding their functional roles during and after brain ischemia remains limited.

Recently, attention has turned to polydendrocytes and their possible role in regeneration following CNS injuries. Polydendrocytes in the adult brain, known as NG2 glia or oligodendrocyte precursor cells, can be identified by their highly branched morphology and their expression of NG2 proteoglycan (NG2) together with platelet-derived growth factor alpha receptor (PDGFαR) [4]. These cells represent a fourth glial population in the mammalian brain, distinct from mature oligodendrocytes, astrocytes or microglia. Until recently they have been assumed to give rise only to oligodendrocytes in the intact adult CNS, although polydendrocytes are known to be capable of generating neurons and astrocytes *in vitro* in the presence of specific morphogens [5], [6] and *in vivo* after transplantation into the hippocampus [7].

Using lineage-specific Cre transgenes, genetic fate-mapping studies in the intact CNS have revealed inconsistent findings. The capability of polydendrocytes to differentiate into oligodendrocytes *in vivo* was clearly confirmed by [8]; however, [9] also described the differentiation of polydendrocytes into grey matter astrocytes. Moreover, several recent studies have described the generation of new neurons in the piriform cortex from polydendrocytes in PDGFαR- or Plp-promoter-driven Cre transgenic mice [8], [10]. On the other hand, in NG2- or Olig2- promoter-driven Cre transgenic animals, such neuronal differentiation was not observed [9], [11].

In CNS pathologies such as global cerebral ischemia, polydendrocytes upregulate NG2 and significantly increase their proliferation rate [12], [13]. Apparently, the pathophysiological changes at the site of injury may enable polydendrocytes to display larger lineage plasticity and thus contribute to CNS regeneration; nevertheless, the electrophysiological properties of polydendrocytes and their fate after CNS injury have not been determined in detail.

In the present study, we examined the fate of polydendrocytes *in vivo* after focal ischemia using transgenic mice that express Cre under the control of the NG2 promoter. We performed complex immunohistochemical and electrophysiological analyses to determine the differentiation potential of polydendrocytes and the electrophysiological properties of the newly-derived cells after ischemia in adult mice.

## Materials and Methods

### Transgenic mice

All procedures involving the use of laboratory animals were performed in accordance with the European Communities Council Directive 24 November 1986 (86/609/EEC) and animal care guidelines approved by the Institute of Experimental Medicine ASCR Animal Care Committee. The generation of NG2creBAC transgenic mice, which express constitutively active Cre in NG2 cells, has been described previously [9]. NG2creBAC mice were crossed with the reporter mouse strain Z/EG to generate NG2creBAC:ZEG double transgenic mice. The mice carrying both modifications were selected by PCR. These double transgenic mice express Cre recombinase under the control of the mouse NG2 (Cspg4) promoter/enhancer. In the Cre recombinase-expressing cells, lacZ expression is replaced with EGFP expression, and these cells can be easily visualized and tracked. Since we detected no sex differences, both male and female mice were used in our experiments. All mouse strains were obtained from The Jackson Laboratory, Bar Harbor, USA.

### Induction of distal middle cerebral artery occlusion (MCAo) in adult mice

Mice (60–90 days old) were anaesthetized for induction with 1.5% Isoflurane and maintained in 1% Isoflurane using a vaporizer (Tec-3, Cyprane Ltd., Keighley, UK). A skin incision between the orbit and the external auditory meatus was made. A 1–2 mm hole was drilled through the frontal bone 1 mm rostral to the fusion of the zygoma and the squamosal bone and about 3.5 mm ventral to the dorsal surface of the brain. The middle cerebral artery (MCA) was exposed after the dura was opened and removed. The MCA was occluded by short coagulation with bipolar tweezers at a proximal location, followed by transection of the vessel to ensure permanent disruption. The mice received 0.5 ml saline subcutaneously, and the body temperature during the surgery was maintained at 37±1°C using a heating pad. Sham operated animals (controls) were subjected to same surgery procedure, without vessel occlusion. To visualize the ischemic region, unfixed brain slices were stained with 2% 2.3.5-triphenyltetrazolium chloride (TTC) at 37°C for 20 minutes.

### Preparation of acute brain slices

The mice were deeply anaesthetized 3, 7 or 14 days after MCAo (3, 7, 14d MCAo) with pentobarbital (PTB) (100 mg/kg, i.p.) and perfused transcardially with cold (4–8°C) isolation solution containing (in mM): 110 NMDG-Cl, 2.5 KCl, 24.5 NaHCO_3_, 1.25 Na_2_HPO_4_, 0.5 CaCl_2_, 7 MgCl_2_, 20 glucose, osmolality 290 mOsm/kg. The mice were decapitated, the brains were quickly dissected out and transversal 200 µm thick slices were cut using a vibration microtome (HM 650 V, Thermo Scientific Microm, Walldorf, Germany). The slices were incubated for 30 minutes at 34°C in the isolation solution and then held at room temperature in artificial cerebrospinal fluid solution (aCSF) containing (in mM): 122 NaCl, 3 KCl, 28 NaHCO_3_, 1.25 Na_2_HPO_4_, 1.5 CaCl_2_, 1.3 MgCl_2_, 10 glucose, osmolality 305 mOsm/kg. Solutions were equilibrated with 95% O_2_/5% CO_2_ to a final pH of 7.4. Osmolality was measured using a vapor pressure osmometer (Vapro 5520, Wescor, Logan, UT, USA).

### Electrophysiological recordings

Acute brain slices were transferred to a recording chamber mounted on the stage of an upright microscope (Axioscop, Zeiss, Gottingen, Germany) equipped with a high-resolution digital camera (AxioCam HRc, Zeiss, Germany) and electronic micromanipulators (Luigs & Neumann, Ratingen, Germany). The chamber was continuously perfused with oxygenated aCSF at a rate of 3 ml/min at 25±2°C. Electrophysiological recordings were performed using an EPC-10 patch-clamp amplifier in combination with PATCHMASTER software (HEKA Elektronik, Lambrecht/Pfalz, Germany). Recording pipettes with a tip resistance of ∼10 MΩ were made from borosilicate capillaries (0.86 ID, Sutter Instruments Company, Novato, CA, USA) using a Brown-Flaming micropipette puller (P-97, Sutter Instruments). Electrodes were filled with an intracellular solution containing (in mM): 130 K-gluconate, 0.5 CaCl_2_, 5 EGTA, 10 HEPES, 3 Mg-ATP and 0.3 Na-GTP; the final pH was adjusted to 7.2 with KOH. The resting membrane potential (RMP) was measured by switching the EPC-10 amplifier to the current-clamp mode. The membrane resistance (Rm) was calculated from the current elicited by a 10 mV test pulse depolarizing the cell membrane from the holding potential of −70 mV to −60 mV for 50 ms, 40 ms after the onset of the depolarizing pulse. Membrane capacitance (Cm) was determined automatically from the Lock-in protocol by PATCHMASTER. Clamping the cell membrane from the holding potential of −70 mV to values ranging from −160 mV to +40 mV for 50 ms at 10 mV intervals evoked membrane currents. To isolate the voltage-gated delayed outwardly rectifying K^+^ (K_DR_) current component, a voltage step from −70 to −60 mV was used to subtract time- and voltage-independent currents. To activate the K_DR_ currents only, the cells were held at −50 mV, and the amplitude of the K_DR_ currents was measured at +40 mV at the end of the pulse. The fast activating and inactivating outwardly-rectifying K^+^ (K_A_) current component was isolated by subtracting the current traces clamped at −110 mV from those clamped at −50 mV, and its amplitude was measured at the peak value. Tetrodotoxin- (TTX, Alomone Labs, Jerusalem, Israel) sensitive Na^+^ currents were isolated by subtracting the current traces measured in 1 µM TTX-containing solution from those measured under control conditions. Na^+^ currents amplitudes were measured at the peak value. Inwardly-rectifying K^+^ (K_IR_) currents were determined at −160 mV at the end of the pulse. Current densities (CD) were calculated by dividing the maximum current amplitudes by the corresponding Cm for each individual cell. The patch-clamp data analyses were performed using FITMASTER software (HEKA Elektronik, Lambrecht/Pfalz, Germany).

### Immunohistochemistry and cell identification

The mice were deeply anaesthetized 3, 7, or 14 days after MCAo with PTB (100 mg/kg, i.p.) and perfused transcardially with 20 ml of saline followed by 20 ml of cooled 4% paraformaldehyde (PFA) in 0.1 M phosphate buffer (PB). Brains were dissected out, post-fixed for 2 hours and placed stepwise in solutions with gradually increasing sucrose concentrations (10%, 20%, 30%) for cryoprotection. Coronal, 30 µm thick slices were prepared using a microtome (HM550, Microm International, Walldorf, Germany). For cell identification after patch-clamp recording, the measured cells were filled with Alexa Fluor 594 hydrazide (0.1 mM; Molecular Probes, Invitrogen, CA, USA) by dialyzing the cytoplasm with the patch pipette solution. Post-recording, the slices were fixed with 4% PFA in 0.1 M PB for 25 minutes and then kept at 4–8°C in phosphate-buffered saline (PBS). The slices were incubated with 5% Chemiblocker (Millipore, MA, USA) and 0.2% Triton in PBS. This blocking solution was also used as the diluent for the antisera. The slices were incubated with the primary antibodies at 4–8°C overnight, and the secondary antibodies were applied for 2 hours. The list of primary and secondary antibodies is summarized in Table 1. To visualize the cell nuclei, the slices were incubated with 300 nM 4, 6-diamidino-2-phenylindole (DAPI) in PBS for 5 minutes at room temperature and mounted using Aqua Poly/Mount (Polysciences Inc., Eppelheim, Germany). All chemicals were purchased from Sigma–Aldrich (St. Louis, MO, USA), unless otherwise stated.

**Table 1:** Primary and secondary antibodies used for immunohistochemistry.

Antigen	Dilution	Isotype	Manufacturer	Secondary antibody
Aldh1	1∶100	Mouse IgG	NeuroMab	GAM 594/660
APC	1∶200	Mouse IgG	Calbiochem	GAM 594/660
Caspase-3	1∶300	Mouse IgG	Alexis Biochem.	GAM 660
CD11b	1∶200	Mouse IgG	Millipore	GAM 594
DCX	1∶1000	Rabbit IgG	Abcam	GAR 594/660
GFAP	1∶800	Mouse IgG	Sigma-Aldrich	Cy3 conjugated
GFP	1∶800	Goat IgG	Abcam	FITC conjugated
GLAST	1∶500	Rabbit IgG	Abcam	GAR 594/660
Ki-67	1∶1000	Rabbit IgG	Abcam	GAR 594/660
Nestin	1∶800	Mouse IgG	Millipore	GAM 594/660
Neun	1∶100	Mouse IgG	Millipore	GAM 594
NG2	1∶400	Rabbit IgG	Millipore	GAR 594/660
Olig-2	1∶1000	Rabbit IgG	Millipore	GAR 660
Pax-6	1∶300	Rabbit IgG	Covance	GAR 594
PCNA	1∶500	Mouse IgG	Abcam	GAM 594
PDGFαR	1∶200	Rabbit IgG	Santa Cruz	GAR 594/660
PDGFβR	1∶200	Rabbit IgG	Santa Cruz	GAR 594/660
S100β	1∶150	Mouse IgG	Sigma-Aldrich	GAM 660
Vimentin	1∶1000	Mouse IgG	Abcam	GAM 594

Aldh1, aldehyde dehydrogenase–1; APC adenomatous polyposis coli; CD11b, integrin alpha M; DCX, doublecortin; GFAP, glial fibrillary acidic protein; GFP, green fluorescent protein; GLAST, glutamate/aspartate transporter; NG2, chondroitin sulfate proteoglycan; PCNA, proliferating cell nuclear antigen; PDGFαR, platelet-derived growth factor receptor alpha; PDGFβR, platelet-derived growth factor receptor beta; S100β, β-subunit of S100 calcium binding protein; GAR 594/660, goat anti-rabbit IgG conjugated with Alexa Fluor 594 or 660; GAM 594/660, goat anti-mouse IgG conjugated with Alexa Fluor 594 or 660 (all secondary antibodies from Invitrogen).

A Zeiss 510DUO LSM confocal microscope equipped with Arg/HeNe lasers and 40× or 63× oil objectives was used for immunohistochemical analysis. Stacks of consecutive confocal images taken at intervals of 3 µm were acquired sequentially with two lasers to avoid cross-talk between fluorescent labels. The background noise of each confocal image was reduced by averaging four image inputs. For each image stack the gain and detector offset were adjusted to minimize saturated pixels, yet still permit the detection of weakly stained cell processes. Colocalization images and their maximum *z* projections were made using a Zeiss LSM Image Browser.

### Cell counts

To determine the fate of polydendrocytes in the peri-lesional cortex, confocal images (315 µm×315 µm×20 µm) covering the borders of the ischemic core were taken from brain coronal slices from control mice and from mice 3, 7 and 14 days after MCAo (5–7 animals from each group, 5 regions from one slice, bregma −1.2 mm) and stained for NG2, nestin, glial fibrillary acidic protein (GFAP), Ki-67 and doublecortin (DCX). The total number of EGFP positive (EGFP^+^) cells and the number of cells that were double positive for EGFP and NG2, nestin, Ki-67, GFAP or DCX were counted in the cortex within the astrogliotic region. Between 1000 and 1500 EGFP^+^ cells were scored. The number of cells was estimated from superimposed images using a GSA Image Analyzer v3.7.7 (Bansemer & Scheel GbR, Rostock, Germany) and expressed as the percentage of double-positive cells from the total number of EGFP^+^ cells.

### Statistics

The results are expressed as the mean ± SEM. Statistical analyses of the differences between groups were performed using one-way ANOVA for multiple comparisons with Dunnett's post-hoc test. Values of p<0.05 were considered significant (*), p<0.01 very significant (**) and p<0.001 extremely significant (***).

## Results

### Under physiological conditions EGFP expression is restricted to polydendrocytes and oligodendrocytes in the dorsal cortex of NG2creBAC:ZEG mice

To determine the fate of polydendrocytes *in vivo*, we used a transgenic mouse strain in which Cre recombinase is expressed under the control of the mouse NG2 promoter [9]. After crossing with Z/EG reporter mice, the NG2creBAC:ZEG mice exhibited widespread EGFP expression in a large population of cells in the brain and spinal cord. Polydendrocytes express NG2 and PDGFαR [14]; however, both proteins are downregulated immediately upon differentiation. Therefore, our approach enabled us to follow the fate of differentiating polydendrocytes based on their EGFP labeling.

**Figure 1:**
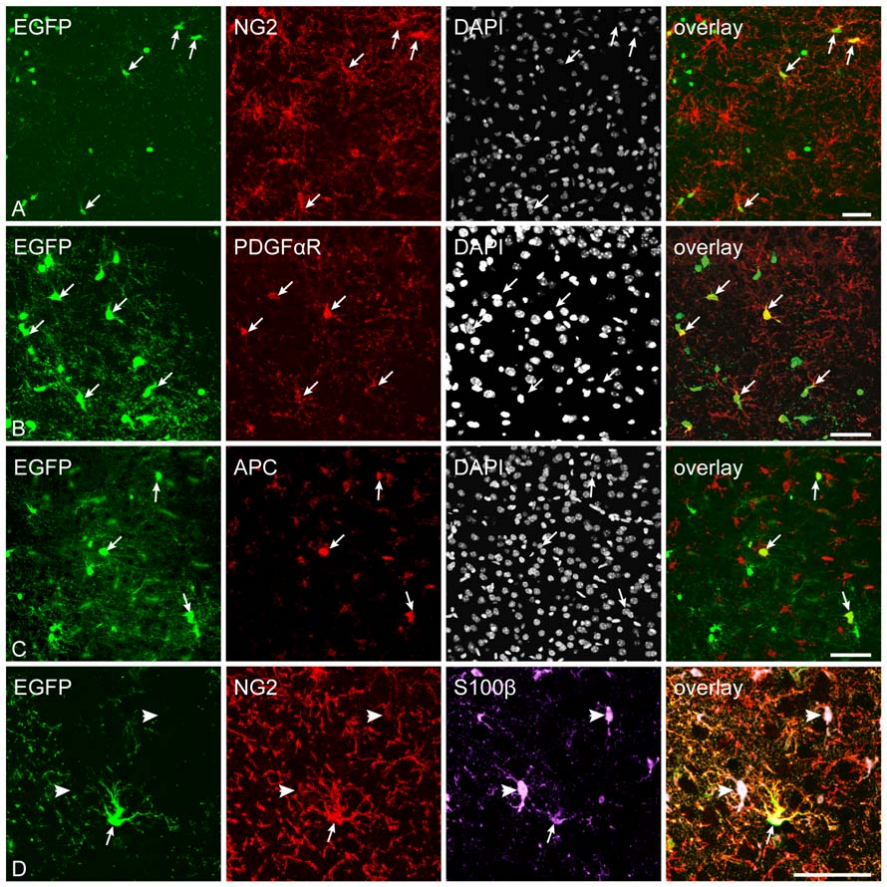
Enhanced green fluorescent protein (EGFP) expression is restricted to polydendrocytes and oligodendrocytes in the control dorsal cortex of NG2creBAC:ZEG mice. ***A***, Immunostaining for NG2 proteoglycan in the cortex illustrating the high number of EGFP^+^/NG2^+^ cells. White arrows highlight several examples. ***B***, The same population of EGFP^+^ cells expressed platelet-derived growth factor alpha (PDGFαR) in a typical polarized pattern only in part of the processes. ***C***, A subpopulation of EGFP^+^ cells in the dorsal cortex expressed the oligodendrocyte lineage marker adenomatous polyposis coli (APC). ***D***, Double immunohistochemistry revealed the co-expression of β-subunit of S100 calcium binding protein (S100β) with NG2 in EGFP^+^ cells, which excludes S100β as a specific marker of astrocytes. The EGFP^−^/NG2^−^ cells are astrocytes (indicated by arrowheads). Scale bars, 50 µm.

To determine the phenotype of EGFP^+^ cells in the adult brain, we performed a complex immunohistochemical analysis of brain coronal sections; first, we counted the total number of EGFP^+^ cells in the dorsal part of the cortex of the control animals. We found 9892±783 EGFP^+^ cells per mm^3^, which represented 4.1%±0.3% of all DAPI^+^ cells. EGFP^+^ cells could be divided into several morphological types. The first, most common morphological phenotype comprised cells with several highly branched processes and small round cell bodies, characteristics typical of polydendrocytes. These cells were predominantly positive for the polydendrocytic markers NG2 and PDGFαR (Fig. 1A, B). NG2 proteoglycan was uniformly expressed on the surface of the cell body and processes; however, staining for PDGFαR revealed its typical polarized expression [8]. EGFP was expressed in 89%±2.3% of NG2^+^ cells throughout the brain, while 55.4%±1.2% of EGFP^+^ cells expressed NG2 in the cortex of control mice, which is in agreement with previous studies performed on this mouse strain [9], [15]. The lack of EGFP detection in approximately 11% of NG2^+^ cells may have been caused by the lack of Cre expression or incomplete Cre-mediated recombination in NG2^+^ cells, or by the inadequate expression of the reporter driven by the promoter in Z/EG mice. NG2^+^ cells are known to possess the highest proliferation rate in the adult mammalian brain, which was confirmed by immunostaining for Ki-67 (a nuclear cellular marker of proliferation). In the dorsal cortex, 3.7%±1.3% of EGFP^+^ cells expressed Ki-67 and 20.6%±2.3% of EGFP^+^ cells expressed PCNA, which is in agreement with previously published data [13], [16]. This discrepancy between the numbers of Ki-67- and PCNA-positive EGFP^+^ cells is probably caused by the longer-lasting stability of PCNA when compared to Ki-67 [17].

The second group of EGFP^+^ cells displayed a typical oligodendrocyte morphology, with large, round cell bodies; they expressed the oligodendrocytic marker Adenomatous polyposis coli (APC, Fig. 1C) and represented 27.6±3.5% of the total number of EGFP^+^ cells. The third group of EGFP^+^ cells in the dorsal cortex had a morphology resembling that of pericytes and, accordingly, they expressed the marker of pericytes – platelet-derived growth factor beta receptor (PDGFβR) – on their cytoplasmatic membrane (Fig. S1A).

Since it has been shown that in some Cre transgenics a certain subpopulation of astrocytes can be EGFP^+^ [11], [18], we analyzed the dorsal cortex for the expression of several astrocytic markers. Nonetheless, we did not detect EGFP^+^ cells expressing any astrocytic markers, such as GFAP, glutamate/aspartate transporter (GLAST) or aldehyde dehydrogenase-1 (Aldh1). However, a subpopulation of EGFP^+^ cells expressed the β-subunit of S100 calcium binding protein (S100β, Fig. 1D). Since S100β is not a specific astrocytic marker and is expressed in astrocytes as well in polydendrocytes [10], [19], we cannot describe these cells as astrocytes. Moreover, these EGFP^+^/S100β^+^ cells expressed NG2 and had the typical morphology of polydendrocytes (Fig. 1D), thus classifying them as polydendrocytes. Finally, we also tested whether some EGFP^+^ cells expressed markers of microglia, but we never detected any EGFP^+^ cells positive for integrin alpha M (CD11b) or Iba-1 (Fig. S1B) – commonly used markers of microglia. Based on our extensive immunohistochemical analysis of the uninjured adult dorsal cortex, we can conclude that the expression of EGFP is restricted only to polydendrocytes, oligodendrocytes and pericytes in adult NG2creBAC:ZEG double transgenic mice.

### Increased proliferation of polydendrocytes in response to focal cerebral ischemia

**Figure 2:**
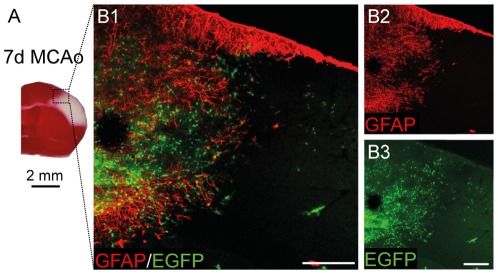
Focal cerebral ischemia leads to a marked increase in the number of EGFP^+^ cells around the ischemic lesion 7 days after MCAo. ***A***, A coronal brain section stained with triphenyltetrazolium chloride indicates the volume of ischemic tissue (white color) 7 days after MCAo. The boxed area in ***A*** is shown in higher magnification in ***B***. ***B1–B2***, An overview image showing glial fibrillary acidic protein (GFAP) overexpression around the ischemic region. ***B3***, Typical localization and a high number of enhanced green fluorescent protein positive (EGFP^+^) cells around the ischemic core 7 days after MCAo. Note that few EGFP^+^ cells were found directly in the ischemic tissue. Scale bars, 50 µm.

To determine whether polydendrocytes in the dorsal cortex of adult mice display an extended differentiation potential in response to ischemic injury, we took advantage of NG2creBAC:ZEG double transgenic mice to enable us to follow cells with NG2 expression even after its loss. The same mouse strains were recently used in a study that described the differentiation of polydendrocytes after a stab wound injury [15] and simultaneously, this study confirmed the suitability of this mouse strain for use in these types of experiments. In contrast to this study we used a highly reproducible model of mouse MCAo with a minimal mortality rate and high reproducibility [20]. Three days after MCAo the ischemic lesion developed in the dorsal brain and occupied approximately one-third of the cortex in the left hemisphere (Fig. 2A). The necrotic tissue was surrounded by massive astrogliosis beginning 3 days after MCAo with a maximal intensity of GFAP staining 7 days after MCAo (Fig. 2B). The size of the ischemic lesion and the intensity of GFAP staining gradually decreased starting on the seventh day of ischemia, and 14 days after MCAo the lesion comprised approximately 25% of its original size.

**Figure 3:**
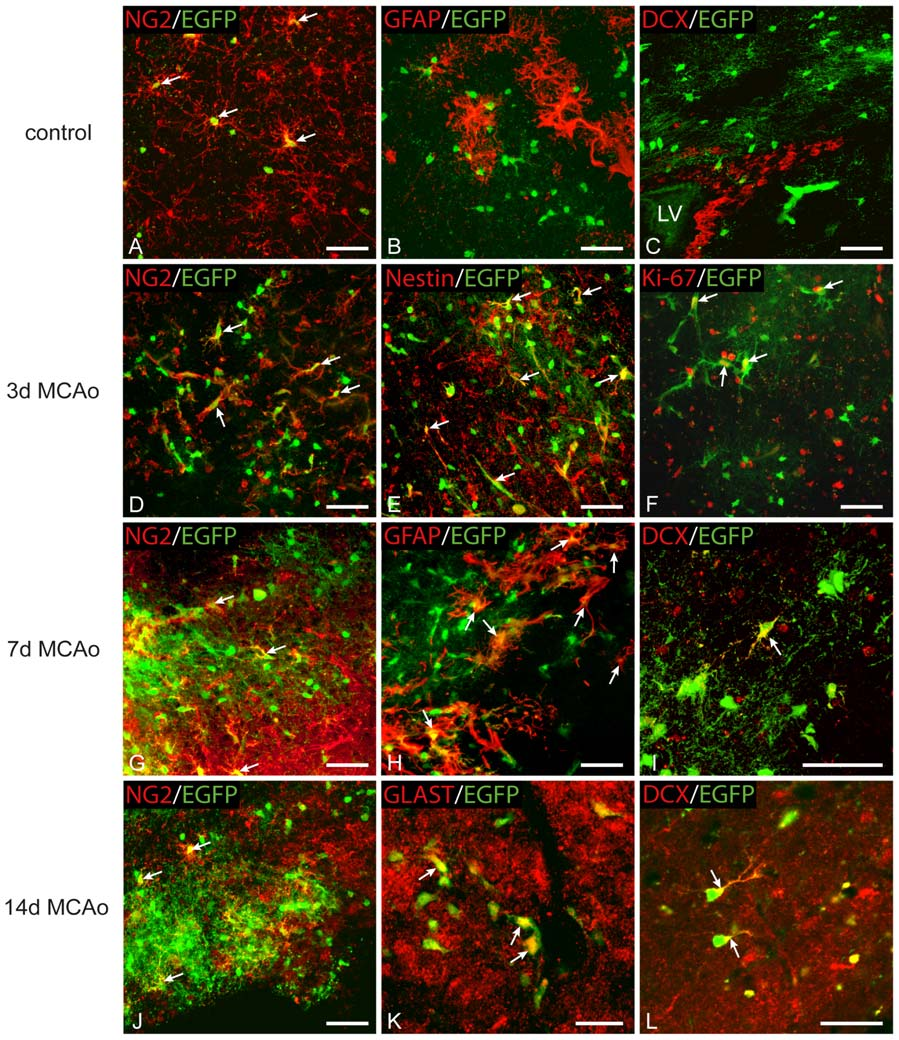
Enhanced green fluorescent protein positive (EGFP^+^) cells express markers of astrocytes and neuronal precursor cells in the dorsal cortex after ischemia. ***A***, EGFP^+^ cells in sham-operated mice (control) expressed a high level of NG2 proteoglycan in the majority of processes. White arrows highlight several examples. ***B***, Virtually no EGFP^+^ cells in the dorsal control cortex were positive for glial fibrillary acidic protein (GFAP). ***C***, Low density of EGFP^+^ cells in the subventricular zone (SVZ). EGFP^+^ cells were never labeled for doublecortin (DCX) in the SVZ of control brains or those after ischemia, which indicates the absence of polydendrocytes from the process of adult neurogenesis; LV, lateral ventricle. ***D***, Three days after MCAo the number of EGFP^+^ cells is significantly increased when compared to controls. Moreover, a large number of EGFP^+^ cells expressed markers of proliferating cells – nestin (***E***) and Ki-67 (***F***). ***G***, EGFP^+^ cells were present at high density seven days after MCAo; however, only a small subpopulation of them expressed NG2 proteoglycan. ***H***, Some EGFP^+^ cells were positive for the astrocytic marker GFAP. ***I***, Starting 7 days after MCAo some scattered cells expressed DCX in their cell bodies and processes. ***J***, Fourteen days after MCAo only a small subpopulation of EGFP^+^ cells expressed NG2 proteoglycan. ***K***, EGFP^+^ cells expressing glutamate/aspartate transporter (GLAST), a marker of mature astrocytes. ***L***, A subpopulation of EGFP^+^ cells displaying polarized and highly developed processes, which were positive for DCX. Scale bars, 50 µm.

**Figure 4:**
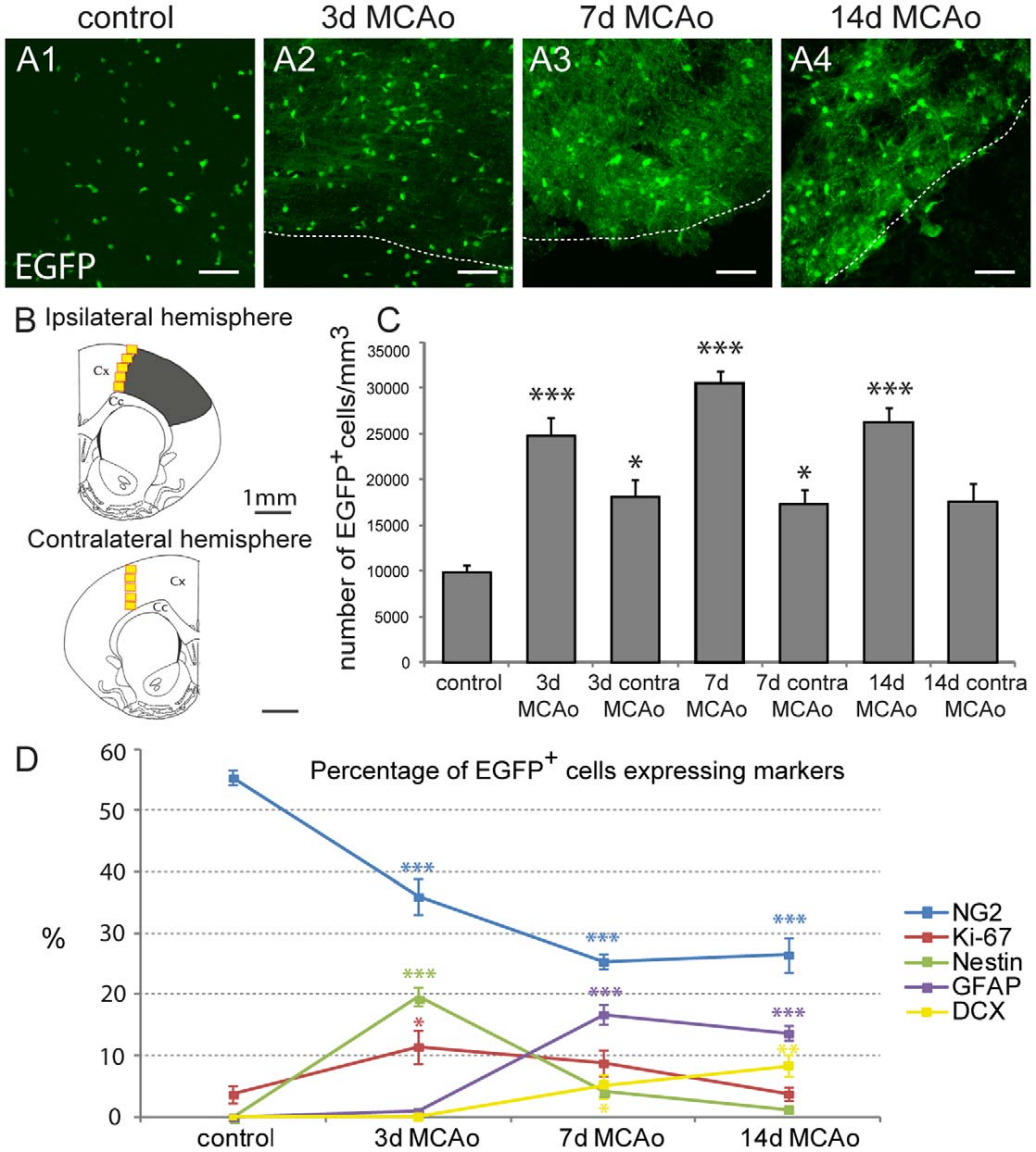
Quantification of the changes in the number of enhanced green fluorescent protein positive (EGFP^+^) cells and in the expression of markers typical for glia, neurons and proliferating cells evoked by MCAo. ***A***, Brain sections showing the increasing number and different distribution of EGFP^+^ cells 3, 7 and 14 days after MCAo (***A2, A3, A4***) when compared to controls (***A1***). Borders of the ischemic tissue are highlighted with the dashed lines. Scale bars, 50 µm. ***B***, Schematic figure of coronal slices of the ipsilateral and contralateral hemispheres depicting the size and localization of tissue injury 7 days after MCAo (grey region), together with the highlighted regions used for immunohistochemical quantification (yellow squares) in both hemispheres; Cx, cortex; Cc, corpus callosum. ***C***, Bar graph showing the quantification of EGFP^+^ cell numbers per 1 mm^3^ in the ipsilateral and contralateral hemispheres in control mice and after MCAo. ***D***, Graph showing the percentage of EGFP^+^ cells expressing markers of polydendrocytes (NG2), proliferating cells (Ki-67, nestin), astrocytes (glial fibrillary acidic protein, GFAP) and neuronal precursor cells (doublecortin, DCX) in controls and after MCAo. Asterisks indicate significant differences between control cortex and post-ischemic cortex; *, p<0.05; ***, p<0.001.

Three days after MCAo the number, distribution and morphology of EGFP^+^ cells were significantly changed at the lesion border (Fig. 3D–F) when compared to controls (Fig. 3A–C). EGFP^+^ cells had larger cell bodies with a smaller number of processes, and their numbers significantly increased (24798±1937 EGFP^+^ cells/mm^3^; p<0.001). The estimated number represented 9.8%±0.6% of all DAPI^+^ cells and was approximately 2.5-fold higher than that found in controls (Fig. 4A2, C). At this time point only 36.0%±2.9% of EGFP^+^ cells expressed NG2 (Fig. 3D, 4D), while in 19.6%±1.5% of EGFP^+^ cells we detected the expression of nestin, which is an intermediate filament upregulated in immature proliferating glia [21] (Fig. 3E). The number of EGFP^+^/nestin^+^ cells was significantly increased when compared to controls (Fig. 4D; p<0.001). Furthermore, the number of proliferating cells, which were positive for Ki-67, significantly increased 3 days after MCAo (Fig. 3F), reaching 11.5%±2.7% of all EGFP^+^ cells (Fig. 4D; p<0.05) and PCNA was expressed in 59.8%±2.5% of EGFP^+^ cells (Fig. S2A). Since recent studies [22] have described the very rapid proliferation of polydendrocytes after injury, we also examined the number of proliferating EGFP^+^ cells in the very early stage after MCAo. Two days after MCAo, 34.9% of EGFP^+^ cells expressed Ki-67 and 75.5% of EGFP^+^ cells expressed PCNA. Only in a few EGFP^+^ cells (6 from 990 EGFP^+^ cells) did we detect a very low expression of GFAP in the cell processes 3 days after MCAo.

Collectively, 3 days after MCAo a large number of polydendrocytes expressed proliferative markers, which accords well with the higher number of EGFP^+^ cells at the lesion borders. In addition, a large number of EGFP^+^ cells appeared that did not express polydendrocytic markers, which might already indicate changes in their phenotype in response to ischemia.

### Polydendrocytes differentiate into reactive astrocytes and neuronal precursor cells in the later stages after ischemia

To follow the fate of polydendrocytes and their progeny during the later stages of astrogliosis, we analyzed brains 7 days after MCAo. At this time point the number of EGFP^+^ cells significantly increased when compared to controls. These EGFP^+^ cells had a more complex morphology with multiple processes, and there was a sharp interface between the ischemic/necrotic and astrogliotic tissue. We counted 30555±1355 EGFP^+^ cells/mm^3^, which represented 12.5%±0.4% of all DAPI^+^ cells (p<0.001). This number was 3-fold higher than in controls (Fig. 4A3, C). However, only 25.4%±1.2% of these EGFP^+^ cells expressed NG2 (Fig. 3G, 4D). Moreover, a large number of newly derived cells started to express proteins that are typical for cell types other than polydendrocytes or oligodendrocytes. Almost 17% (16.8%±1.6%) of EGFP^+^ cells expressed high levels of GFAP in their processes (Fig. 3H, 4D, S2B), and these GFAP^+^/EGFP^+^ cells were localized in regions very close to the ischemic tissue or had even migrated directly into the ischemic core (Fig. 3H). These cells lacked the typical bushy protoplasmic morphology but resembled the morphology of reactive astrocytes, and some of them were positive for markers of reactive astrocytes, such as vimentin (data not shown). The expression of nestin in EGFP^+^ cells declined 7 days after MCAo: nestin^+^/EGFP^+^ cells comprised 4.3%±1.0% of all EGFP^+^ cells (Fig. 4D). However, nestin expression did not entirely correlate with GFAP staining in EGFP^+^ cells and was still present in EGFP^+^/NG2^+^ cells. Furthermore, a small subpopulation of GFAP^+^/EGFP^+^ cells expressed Ki-67 (Fig. S2C) while in total 8.8%±2.1% of EGFP^+^ cells were positive for this marker (Fig. 4D).

Since it has been demonstrated previously that polydendrocytes can give rise to neurons *in vitro* and after their transplantation into the hippocampus [7], [23], we examined EGFP^+^ cells for the expression of DCX, a marker of newly derived neurons and neuroblasts. Surprisingly, we found that almost 5.0%±1.9% of EGFP^+^ cells (Fig. 4D) expressed DCX 7 days after MCAo. The expression of DCX was localized in the cell bodies as well as in the short processes (Fig. 3I, S2D). Additionally, we also found the expression of the transcription factor Pax-6 in several EGFP^+^ cells 3 and 7 days after MCAo (Fig. S3A). This transcription factor is known to drive glial cells to adopt a neuronal phenotype [24]. To exclude the possibility that DCX^+^/EGFP^+^ cells in the ischemic border might originate from the neurogenic region in the subventricular zone (SVZ), we also analyzed in detail the expression of EGFP in this area. In agreement with previously published studies [10], [25], we never observed the co-expression of EGFP with other than polydendrocytic markers in the SVZ and, moreover, the density of EGFP^+^ cells in the lateral part of the SVZ was lower than in the surrounding nervous tissue (Fig. 3C). Moreover, we also tested if EGFP^+^ cells expressed some markers of reactive microglia after MCAo; however, we never detected any expression of CD11b (Fig. S3B) or Iba-1 in EGFP^+^ cells (Fig. S3C).

Further analyses of ischemic brains 14 days after MCAo revealed that the number of EGFP^+^ cells was still significantly enhanced when compared to controls (26199±1763 cells/mm^3^) and that they represented 9.3%±0.4% of all DAPI^+^ cells (Fig. 4A4, C; p<0.001). Immunohistochemical staining for NG2 revealed its high expression at the lesion border, but only 26.5%±2.8% of EGFP^+^ cells expressed this proteoglycan (Fig. 3J). The number of GFAP^+^/EGFP^+^ cells was slightly decreased compared to 7 days after MCAo, but still 13.7%±1.2% of EGFP^+^ cells expressed GFAP (Fig. 4D). Furthermore, these cells seemed to have a more developed phenotype, based on their strong expression of the astrocyte-specific marker GLAST (Fig. 3K). The number of proliferating EGFP^+^ cells declined and reached the proliferation rate of the controls, and only 1.2%±0.4% of EGFP^+^ cells expressed nestin (Fig. 4D). On the other hand, we still detected a large number of DCX^+^/EGFP^+^ cells comprising 8.9%±1.6% of all EGFP^+^ cells (Fig. 4D). Concurrently, in these cells immunohistochemistry revealed the localization of DCX protein predominantly in the processes, which were more developed than those observed 7 days after MCAo (Fig. 3L).

In brain sections obtained one month after MCAo we detected only rarely the co-expression of EGFP with markers of mature neurons. There were only a few EGFP^+^/NeuN^+^ cells in the vicinity of the ischemic lesion (data not shown); however, we also found several DCX^+^/EGFP^+^ cells expressing the apoptotic marker caspase-3 (Fig. S3D).

In addition, we examined brain sections from all time points for the oligodendrocytic marker APC; however, the number of APC^+^/EGFP^+^ cells was very low in the proximity of the ischemic region, therefore immunohistochemical quantification was not carried out.

In summary, a detailed analysis of the ischemic border between 7 and 30 days after MCAo revealed that polydendrocytes can contribute to the generation of reactive astrocytes and, moreover, they can differentiate into a transient population of immature neuronal precursor cells, probably due to the influence of growth factors near the ischemic tissue.

### Polydendrocytes do not change phenotype in the contralateral hemisphere of ischemic animals

Concurrently, we examined the contralateral uninjured hemisphere for the number of EGFP^+^ cells and the expression of the studied markers at all time points after MCAo. Three days after MCAo we found 18066±1951 EGFP^+^ cells/mm^3^, which represented 7.3%±0.8% of all DAPI^+^ cells in the dorsal contralateral cortex. This number was significantly higher compared to controls, and the increased quantity of EGFP^+^ cells was maintained 7 days after MCAo as well (Fig. 4C; p<0.05). To determine the phenotype of the EGFP^+^ cells in the contralateral hemisphere, we analyzed the brain sections for all of the studied markers. We found no significant changes in the expression of cell type-specific markers in EGFP^+^ cells compared to controls with the exception of proliferation markers (Ki-67, PCNA, nestin). We found that Ki-67 was expressed by 9.2±2.8% of all EGFP^+^ cells 3 days after MCAo, which was a significantly higher percentage than that found in controls (p<0.05). Interestingly another proliferation marker, PCNA, was expressed by 51.9% of EGFP^+^ cells. Moreover, some EGFP^+^ cells expressing low levels of nestin were found in the contralateral hemisphere 3 days after MCAo (data not shown).

### Polydendrocytes respond to ischemia with significant changes in their electrophysiological properties

Based on immunohistochemical staining, we found that polydendrocytes, besides their self-renewal, give rise to a large population of astrocytes, but also to neuronal precursor cells. Next, we were interested to determine what electrophysiological properties polydendrocytes and their progeny display after MCAo.

**Figure 5:**
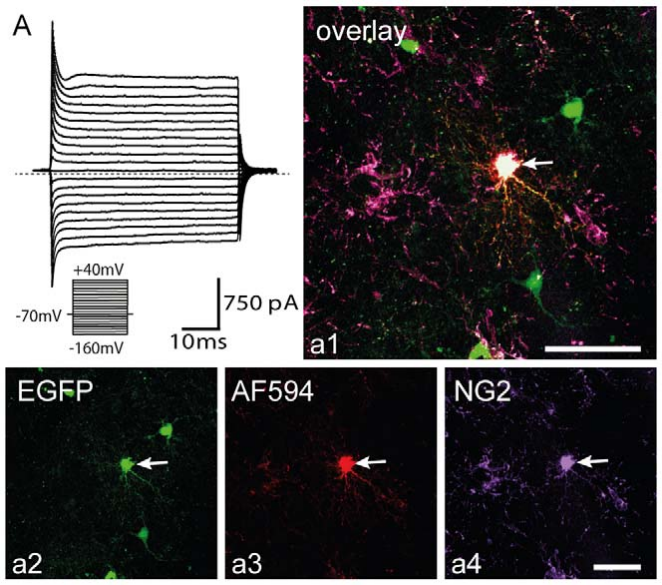
Enhanced green fluorescent protein positive (EGFP^+^) cells in the cortex of sham-operated mice are NG2^+^ polydendrocytes displaying a complex current pattern. ***A***, Polydendrocytic current pattern in controls measured after depolarizing the cell membrane from a holding potential of −70 mV to +40 mV and hyperpolarizing to −160 mV (see the inset, bottom). Polydendrocytes showed a complex current pattern, i.e., time- and voltage-independent K^+^ currents, K_DR_, K_A_ and K_IR_ currents. The dashed line marks zero current. ***a1–a4***, Polydendrocyte expressing EGFP in control cortex (***a2***), loaded with Alexa Fluor 594 hydrazid during patch-clamp measurement (***a3***), subsequently stained with an antibody directed against NG2 proteoglycan (***a4***). Scale bars, 50 µm.

**Figure 6:**
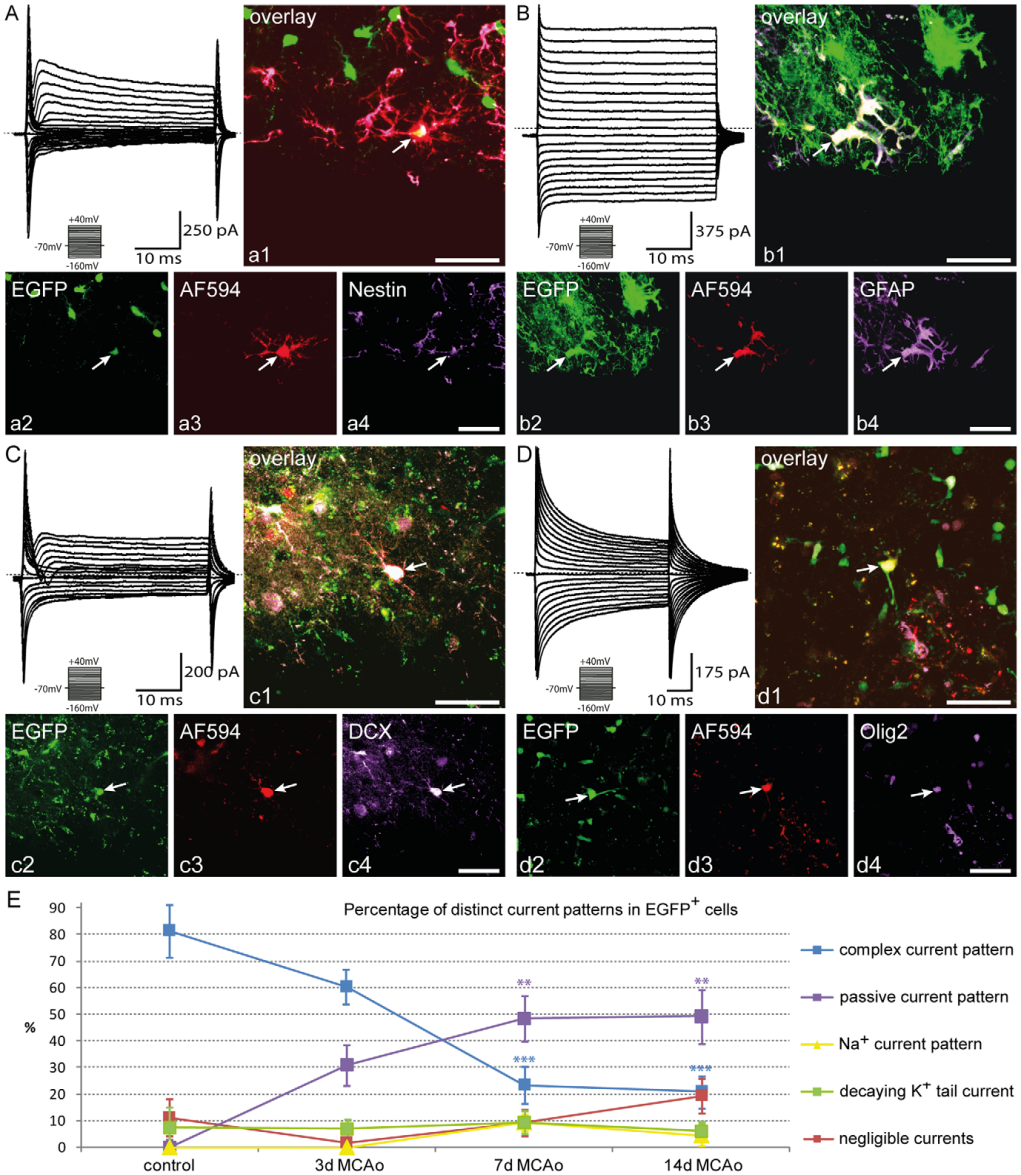
Polydendrocytes differentiate into cells with distinct current patterns after MCAo. ***A***, A typical current pattern of polydendrocytes measured 3 days after MCAo in response to cell membrane depolarization from a holding potential of −70 mV to +40 mV and hyperpolarization to −160 mV (see the inset, bottom). Polydendrocytes showed a current pattern with K_DR_, K_A_, small K_IR_ currents and fast activating Na^+^ inward currents in a few cells 3 days after MCAo. Zero current is marked by the dashed line. ***a1–a4***, Polydendrocyte expressing enhanced green fluorescent protein (EGFP) 3 days after MCAo (***a2***) loaded with Alexa Fluor 594 hydrazid during patch-clamp measurement (***a3***) subsequently stained with an antibody directed against nestin (***a4***). White arrow indicates the recorded cell. Scale bars, 50 µm. ***B***, Current pattern of EGFP^+^ astrocytes 7 days after MCAo recorded using the same protocol as in (***A***). EGFP^+^ astrocytes showed a passive current pattern, i.e., time- and voltage-independent non-decaying K^+^ currents, with only small voltage activated K^+^ currents. Zero current is marked by the dashed line. ***b1–b4***, Astrocytes expressing EGFP 7 days after MCAo (***b2***) loaded with Alexa Fluor 594 hydrazid during patch-clamp measurement (***b3***), subsequently stained with an antibody directed against glial fibrillary acidic protein (GFAP, ***b4***), a marker of astrocytes. Note dye coupling (b3) between EGFP^+^ cells with a passive current pattern compared to EFGP^+^ cells with a complex current pattern in A (a3). Scale bars, 50 µm. ***C***, Current pattern of EGFP^+^ neuronal precursor cells 7 days after MCAo measured with the same protocol as in (***A***). EGFP^+^ neuronal precursor cells displayed outwardly rectifying K^+^ currents (K_DR_, K_A_ currents) together with higher Na^+^ currents when compared to polydendrocytes. ***c1–c4***, Neuronal precursor cells expressing EGFP 7 days after MCAo (***c2***), loaded with Alexa Fluor 594 hydrazid during patch-clamp measurement (***c3***), subsequently stained with an antibody directed against doublecortin (DCX, ***c4***), a marker of newly-derived neuronal precursor cells. Scale bars, 50 µm. ***D***, Current pattern of EGFP^+^ oligodendrocytes 7 days after MCAo measured with the same protocol as in (***A***). EGFP^+^ oligodendrocytes displayed time- and voltage-independent K^+^ currents decaying during the duration of the voltage pulse. ***d1–d4***, Oligodendrocytes expressing EGFP 7 days after MCAo (***d2***) loaded with Alexa Fluor 594 hydrazid during patch-clamp measurement (***d3***), subsequently stained with an antibody directed against Olig2 (***d4***). **E**, Graph showing the percentage of EGFP^+^ cell with distinct current patterns. Asterisks indicate significant differences between control cortex and post-ischemic cortex; **, p<0.01; ***, p<0,001.

Using the patch-clamp technique in the whole cell configuration, we initially determined the electrophysiological phenotype of EGFP^+^ cells in control brains. Intracellular staining with a fluorescent dye revealed that many EGFP^+^ cells had several radially oriented primary and secondary processes, resembling the multi-process morphology typical of polydendrocytes, as described previously [26]. Post-recording immunohistochemical analysis confirmed that these EGFP^+^ cells are positive for PDGFαR and NG2 (Fig. 5a1). In agreement with previous findings [27], we never observed dye-coupling between individual NG2^+^/EGFP^+^ cells in controls or after MCAo. As expected, electrophysiological recordings revealed a complex current pattern typical of a polydendrocytic population, i.e., they displayed time- and voltage-independent K^+^ conductance together with K_DR_, K_A_ and K_IR_ currents in controls [28] (Fig. 5A). Their average RMP was −77.5±1.3 mV (n = 22), Rm was 121.1±10.7 MΩ and Cm was 9.5±0.8 pF. Current densities were 17.5±6.5 pA/pF for K_IR_ currents, 8.1±2.3 pA/pF for K_DR_ currents and 12.4±3.0 pA/pF for K_A_ currents (Table 2.). Cells displaying a complex current pattern represented 81.5%±9.9% of all measured EGFP^+^ cells in the control cortex (Fig. 6E).

**Table 2:** Electrophysiological properties of EGFP^+^ cells with a complex current pattern.

	Control	3 d MCAo	7 d MCAo	14 d MCAo
RMP (mV)	−77.5±1.3	−69.5±2.1 (*)	−59.5±5.2 (***)	−65.2±3.1 (*)
Rm (MΩ)	121.1±10.7	525.3±88.4 (**)	372.7±42.5	309.3±39.7
Cm (pF)	9.5±0.8	8.9±0.8	7.2±0.9	8.8±1.3
K_IR_ (pA/pF)	17.5±6.5	3.3±0.8 (**)	9.6±1.3	7.0±1.3
K_DR_ (pA/pF)	8.1±2.3	29.6±3.4 (***)	31.2±3.6 (***)	8.3±1.9
K_A_ (pA/pF)	12.4±3.0	14.7±2.1	11.7±3.0	14.6±3.4
Na (pA/pF)	0.0±0.0	3.5±1.3 (*)	0.0±0.0	0.0±0.0
n	22	35	15	14
%	81.0±9.9	60.0±6.6	23.4±6.9 (***)	20.9±6.1 (***)

RMP, resting membrane potential; Rm, membrane resistance; Cm, membrane capacitance; K_IR_, current density of inwardly-rectifying K^+^ currents; K_DR_, current density of voltage-gated delayed outwardly-rectifying K^+^ currents; K_A_, current density of the fast activating and inactivating outwardly-rectifying K^+^ currents; Na, current density of Na^+^ currents; n, number of measured cells; %, percentage of the cell type;

* , p<0.05;

** , p<0.01;

*** , p<0.001.

After MCAo we found EGFP^+^ cells with a complex current pattern at all studied time points; however, their number, passive membrane properties and current densities were markedly changed when compared to controls. Three days after MCAo EGFP^+^ cells displaying a complex current pattern had a significantly increased Rm (p<0.01), which accords well with the significantly decreased CD of K_IR_ currents and time- and voltage-independent K^+^ conductance when compared to controls (Table 2.; p<0.01). On the other hand, the CD of K_DR_ currents was significantly increased when compared to controls (p<0.001). These changes in the membrane properties have been shown to reflect the high proliferative activity of polydendrocytes soon after ischemia [13], [29], which is also in good correlation with the increased expression of Ki-67 and nestin in EGFP^+^ cells 3 days after ischemia. Interestingly, in this early phase after ischemia we also detected TTX-sensitive Na^+^ currents with a low CD of 3.6±1.3 pA/pF in these cells (8 from 35 cells, Fig. 6A); their presence probably contributed to the significantly depolarized RMP when compared to controls (p<0.05) [13], [27]. Since Na^+^ currents in grey matter polydendrocytes are detected predominately in the early postnatal stages [27], we might conclude that polydendrocytes early after ischemia resemble the immature developing stages of polydendrocytes [23]. Post-recording immunohistochemical identification 3 days after MCAo revealed their positivity for NG2 and nestin (Fig. 6a1). Seven days after MCAo the percentage of EGFP^+^ cells with a complex current pattern had significantly decreased to 23.4%±6.9% (p<0.001) (Fig. 6E). These cells were depolarized and, moreover, they had significant increased CD of K_DR_ currents when compared to controls (p<0.001). Fourteen days after MCAo we found a further significant decrease in the quantity of the EGFP^+^ cells with a complex current pattern when compared to controls. Only 20.9%±6.1% (p<0.001) of all measured EGFP^+^ cells revealed a complex current pattern (Fig. 6E). These cells had a significantly depolarized RMP (p<0.05), but no other significant differences in their membrane properties were found when compared to controls. These cells always expressed only polydendrocytic markers, such as NG2 and PDGFαR, 7 and 14 days after MCAo.

Taken together, our electrophysiological analyses of polydendrocytes during the post-ischemic period revealed significant changes in their membrane properties 3 days after MCAo that coincided with their increased proliferation and their declining incidence 7 and 14 days after MCAo.

### Polydendrocytes differentiate into cells with the functional properties of astrocytes after ischemia

During the post-ischemic period a subpopulation of EGFP^+^ cells displayed a current profile with predominant high time- and voltage-independent non-decaying K^+^ conductance, a so-called passive current pattern (Fig. 6B). These cells were detected in all post-ischemic periods, and their passive membrane properties as well as their current densities did not significantly differ at the studied time points after MCAo (Table 3.). Fourteen days after MCAo these cells had an RMP of −47.5±1.0 mV, Rm was 135.3±8.0 MΩ and Cm reached 7.5±0.6 pF (n = 33). The current density reached 0.5±0.3 pA/pF for K_IR_ and 8.0±3.3 pA/pF for K_DR_ currents, which was completely blocked by 1 mM 4-aminopyridine (4-AP). We found significant differences in the percentage of these cells in the post-ischemic periods. EGFP^+^ cells with a passive current pattern were never detected in controls, and their distribution increased with time after ischemia. Their number was significantly increased 7 and 14 days after MCAo when compared to controls; they represented 48.4%±8.5% (p<0.01) and 49.3%±10.2% (p<0.01), respectively, of all measured cells (Fig. 6E). Some of them expressed nestin, Ki-67 or GFAP 3 days after MCAo; however, in the later phases after MCAo only GFAP (Fig. 6b1) or GLAST were detected by immunohistochemical post-recording identification. Starting 7 days after MCAo intracellular staining with a fluorescent dye showed that some EGFP^+^ cells with a passive current pattern were dye-coupled to other EGFP^+^ or EGFP^−^ cells (Fig. 6b3). We can conclude that a certain subpopulation of newly-derived cells of polydendrocyte origin expresses astrocytic markers and these cells also display membrane properties typical of reactive astrocytes.

### Polydendrocytes also differentiate into cells with the functional properties of immature neuronal precursor cells after ischemia

In agreement with our immunohistochemical analysis, we also detected a subpopulation of EGFP^+^ cells that expressed a current profile that resembled that of neuronal precursor cells (Fig. 6c). These cells were detected 7 and 14 days after MCAo, and their passive membrane properties as well as the current densities of their voltage activated channels did not significantly differ between these two time points. Seven days after MCAo these cells had an RMP about −58.2±5.0 mV, their Rm was 840.4±144.9 MΩ and Cm was 5.2±0.9 pF (n = 6). The current densities reached 1.3±1.3 pA/pF for K_IR_ currents, 34.7±6.0 pA/pF for K_DR_ currents and 17.9±2.0 pA/pF for K_A_ currents. These cells also expressed TTX-sensitive Na^+^ currents, but with a significantly higher CD (39.5±10.2 pA/pF, p<0.01) (Table 4.) and clearly different kinetics when compared to polydendrocytes with Na^+^ currents 3 days after MCAo. The Na^+^ currents in neuronal precursor cells displayed significantly faster activation after depolarization steps when compared to Na^+^ currents in polydendrocytes and, in addition, these currents were activated at a significantly lower voltage threshold (−18.0±5.8 mV) than in polydendrocytes (2.9±4.2 mV, p<0.05). The percentage of neuronal precursor cells reached 9.4%±3.1% of all measured cells 7 days after MCAo and 4.5%±4.0% 14 days after MCAo (Fig. 6E). Post-recording immunohistochemical identification confirmed DCX expression in EGFP^+^ neuronal precursor cells (Fig. 6c1).

### After ischemia only a small population of oligodendrocytes in the cortex arises from polydendrocytes

Furthermore, in some EGFP^+^ cells we have recorded time- and voltage-independent K^+^ currents decaying during the duration of the voltage pulse, a typical current pattern of oligodendrocytes [30] (Fig. 6D). These cells were present at all analyzed time points, and their electrophysiological properties and quantity did not significantly change after MCAo when compared to controls (Fig. 6E). Seven days after MCAo they had an RMP of −54.5±4.9 mV, Rm was 815.8±120.6 MΩ and Cm was 9.8±3.9 pF (n = 6). The current densities reached 4.4±4.4 pA/pF for K_IR_ currents, 11.6±5.2 pA/pF for K_DR_ currents and 6.5±3.6 pA/pF for K_A_ currents. These cells were positive for APC or Olig2 (Fig. 6d1) as revealed by post-recording immunohistochemical identification.

**Table 3:** Electrophysiological properties of EGFP^+^ cells with a passive current pattern.

	3 d MCAo	7 d MCAo	14 d MCAo
RMP (mV)	−56.0±4.4	−52.8±2.7	−47.5±1.0
Rm (MΩ)	165.0±13.0	168.4±8.3	135.3±8.0
Cm (pF)	8.4±0.9	9.7±1.6	7.5±0.6
K_IR_ (pA/pF)	2.7±1.3	2.8±1.1	0.5±0.3
K_DR_ (pA/pF)	10.5±3.0	9.0±1.9	8.0±3.3
K_A_ (pA/pF)	0.0±0.0	0.0±0.0	0.0±0.0
Na (pA/pF)	0.0±0.0	0.0±0.0	0.0±0.0
n	18	31	33
%	31.0±7.6	48.4±8.5	49.3±10.2

RMP, resting membrane potential; Rm, membrane resistance; Cm, membrane capacitance; K_IR_, current density of inwardly-rectifying K^+^ currents; K_DR_, current density of voltage-gated delayed outwardly-rectifying K^+^ currents; K_A_, current density of the fast activating and inactivating outwardly-rectifying K^+^ currents; Na, current density of Na^+^ currents; n, number of measured cells; %, percentage of the cell type.

### Certain subpopulation of EGFP^+^ cells in the ischemic cortex are pericytes

Finally, the last identified subpopulation based on electrophysiological properties comprised cells with negligible voltage activated or time- and voltage-independent K^+^ currents and was present in both control and post-ischemic brains. These cells did not significantly differ in their electrophysiological properties or quantity (Fig. 6E) at any studied time points. Post-recording immunohistochemical identification showed that they did not express common neuronal or glial markers; however, we were able to occasionally detect PDGFβR expression on their surface, which classified them as pericytes.

## Discussion

In the present study, we used mice with constitutively active Cre driven by the NG2-promoter that, after breeding with a reporter strain, allow fate mapping of a large population of polydendrocytes. In accordance with previous studies [10], we detected the generation of oligodendrocytes in the uninjured adult brain; however, in contradiction to some earlier reports [9], [18], our study did not confirm the generation of astrocytes and therefore excludes polydendrocytes as multipotent cells in the healthy adult brain.

After detailed characterization of the phenotype of EGFP^+^ cells in the healthy brain, the mice were subjected to focal cerebral ischemia, which triggered dramatic changes in the morphology, number and phenotype of EGFP^+^ cells. The number of EGFP^+^ cells after ischemia was significantly increased; the cells displayed heterogeneous morphology and expressed markers of astrocytes and neuronal precursor cells. Moreover, polydendrocyte multipotency was confirmed by detailed electrophysiological analysis, which also proved the generation of astrocytic and neuronal precursor phenotypes from EGFP^+^ cells after ischemia.

### Polydendrocytes are unipotent cells in the healthy adult brain

A number of recent studies have shown the importance of selecting an appropriate polydendrocytic marker as a Cre promoter. In our study we used NG2, as a generally accepted marker of polydendrocytes and, moreover, this proteoglycan is not down-regulated after injury as in the case of PDGFαR [12]. Although NG2 and PDGFαR are strongly co-expressed in control cells, more distinct polydendrocytic subpopulations can arise after ischemia.

We used the NG2creBAC mice strain with constitutively active Cre expression and a high recombination rate, in which a large polydendrocyte population and their progeny express EGFP. This enabled us to analyze a larger sample of EGFP^+^ cells before and after ischemia than in mice with an inducible form of Cre with low recombination efficiency. Although inducible Cre brings the advantage of labeling a cohort of cells with a currently expressed marker, our analysis proved that we had a well characterized cell population clearly restricted to only polydendrocytes, oligodendrocytes and pericytes before ischemia in the adult cortex. Polydendrocytes expressing NG2 and EGFP displayed immunocytochemical and electrophysiological properties that suggested the absence of any additional subpopulations of polydendrocytes in the adult cortex. This finding is in agreement with study [31], where was detected a uniform population of polydendrocytes in the adult hippocampus.

### Polydendrocytes respond to ischemia by increased proliferation

After ischemia we detected a significantly increased number of EGFP^+^ cells on the ischemic lesion edge at all studied time points, a phenomenon already described after a stab wound [11], [15], cryoinjury [32], in experimental autoimmune encephalomyelitis [33] or focal demyelination [34]. Based on the extensive proliferation of polydendrocytes in the early stages after injury (a 3-fold higher number of Ki-67^+^/EGFP^+^ cells and a 2.9-fold higher number of PCNA^+^/EGFP^+^ cells 3 days after MCAo when compared to controls), we assume that this increased number of EGFP^+^ cells is caused by the generation of new EGFP^+^ cells from existing polydendrocytes. However, we cannot entirely exclude polydendrocyte migration from the surrounding tissue to the site of injury. Another eventuality is that other cell types began to transiently express NG2, as was shown, for example, in the case of microglia cells. Although several recent studies have reported the low expression of NG2 in resting microglia, we never detected EGFP in these CD11b^+^/Iba-1^+^ cells in the control brain [35]. Moreover, the transiently increased expression of NG2 has been shown in activated microglia cells after several types of injury [35]–[37]; on the other hand, other studies exclude the possibility of NG2 expression in these cells [31], [38], [39]. In our study, we never detected EGFP^+^ cells that displayed any microglial immunohistochemical or electrophysiological properties at any studied time point after MCAo. This phenomenon indicates that microglia cells after injury really do not activate the NG2 promoter or that a weak or short, transient activation of the CSPG4 promotor in the adult brain is not sufficient to trigger the expression of EGFP in other cells types, thus confirming that newly-derived EGFP^+^ cells originate from pre-ischemic EGFP^+^ cells.

**Table 4:** Electrophysiological properties of EGFP^+^ neuronal precursor cells.

	7 d MCAo	14 d MCAo
RMP (mV)	−58.2±5.0	−62.7±9.3
Rm (MΩ)	840.4±144.9	1189.7±631.9
Cm (pF)	5.2±0.9	6.9±0.9
K_IR_ (pA/pF)	1.3±1.3	0.0±0.0
K_DR_ (pA/pF)	34.70±6.0	46.1±5.8
K_A_ (pA/pF)	17.9±2.0	15.7±2.5
Na (pA/pF)	39.5±10.2	21.5±12.0
n	6	3
%	9.4±3.1	4.5±4.1

RMP, resting membrane potential; Rm, membrane resistance; Cm, membrane capacitance; K_IR_, current density of inwardly-rectifying K^+^ currents; K_DR_, current density of voltage-gated delayed outwardly-rectifying K^+^ currents; K_A_, current density of the fast activating and inactivating outwardly-rectifying K^+^ currents; Na, current density of Na^+^ currents; n, number of measured cells; %, percentage of the cell type.

Immunohistochemical analysis showed an increased amount of NG2 at the lesion site in the later phases after ischemia, which accords well with previously published results [40]; however, the total number of EGFP^+^ cells with detectable NG2 expression decreased. This phenomenon can be explained by the increased release of soluble NG2 form from many cell types into the extracellular space [40] and, concurrently, by differentiating of NG2^+^ polydendrocytes into another cell type.

Genetic fate mapping enabled us to determine the functional properties of polydendrocytes even in the later phases after ischemia, when it is extremely difficult to perform patch-clamp measurements of newly-derived cells in the gliotic scar full of reactive astrocytes and microglia. We found that polydendrocytes responded to ischemia with marked changes in their electrophysiological properties, especially in the early phases after ischemia. However, in the late phases after ischemia their membrane properties were comparable to those of controls, indicating the rapid recovery of polydendrocytic electrophysiological properties to the pre-ischemic state.

### Polydendrocytes display multipotent differentiation potential after ischemia

Based on immunohistochemical analysis, ∼75% of EGFP^+^ cells did not express NG2 14 days after MCAo and electrophysiological analysis revealed ∼80% of EGFP^+^ cells with a current pattern distinct from that of polydendrocytes. The expression of GFAP, GLAST or vimentin together with a passive current pattern and dye-coupling in a large subpopulation of EGFP^+^ cells evidenced that polydendrocytes can give rise to reactive astrocytes after ischemia. The generation of a reactive astrocyte subpopulation from polydendrocytes was already shown in models of cryoinjury [32], stab wound injury, experimental autoimmune encephalomyelitis or focal demyelination. However, in the majority of these injuries the generation of new astrocytes was limited and represented ∼8% of EGFP^+^ cells, and their number declined in the later phases after injury. In our study we detected ∼17% of EGFP^+^ cells with an astrocytic phenotype 7 days after ischemia, and their number only slightly decreased in the later phases after ischemia. This discrepancy is probably caused by using different injury models. In addition, the majority of studies employed rather mild types of cortical injuries or models of demyelination in the spinal cord; in contrast, we used a model of severe ischemic brain injury, in which the stronger involvement of repair processes and higher levels of growth factors could play an important role, resulting in greater differentiation of polydendrocytes into astrocytes. Moreover, we analyzed in detail the electrophysiological properties of newly-derived astrocytes. These cells had a passive current profile with 4-AP sensitive K_DR_ currents, which were further decreased in the later phases after ischemia. Our results are in good agreement with previously published electrophysiological analyses of reactive astrocytes after ischemia in the hippocampus [13] and cortex [41] and show that these newly-derived astrocytes are comparable with other reactive astrocyte populations in the later phases after ischemia.

Importantly, we also identified cells of polydendrocyte origin that displayed the immunohistochemical and electrophysiological properties of neuronal precursor cells and comprised ∼9% of EGFP^+^ cells 7 days after ischemia. Although recent studies described DCX expression and the differentiation of PDGFαR^+^ or PLP^+^ cells into neurons in the healthy adult brain, this phenomenon was always strictly localized only in the piriform cortex [8], [10]. Nevertheless, in our study we did not detect any EGFP^+/^DCX^+^ cells in any brain regions including the SVZ of sham-operated mice. After ischemia, some EGFP^+^/DCX^+^ cells displayed Na^+^ currents with a significantly higher CD and markedly different kinetics when compared to polydendrocytes, together with high membrane resistance, which is a typical feature of immature neuroblasts [42]. Since it is relatively easy to force polydendrocytes *in vitro* to differentiate into neurons [5]–[7], a suitable mixture of growth factors and morphogenes released at the lesion site can probably also promote this phenomenon *in vivo*.

However, detailed analysis of these cells in the later phases after ischemia revealed that these cells were probably incapable of surviving and maturing into cells with a more developed neuronal phenotype. This assumption is based on our observation of the strong expression of caspase-3 in several EGFP^+^/DCX^+^ cells, and their inability to survive in the damaged ischemic region was also described previously in the newly-derived SVZ neuroblasts migrating into the site of injury after stroke [43]. We described the generation of neuronal precursor cells in mature, but still very young mice; however, we must assume that multipotency and the ability to differentiate into different cell types is strongly correlated with age. Many studies describing the multipotency of polydendrocytes were performed using postnatal or very young animals [5], [6], so the ability to produce cells of the neuronal lineage could be strongly suppressed in older mice. The same scenario probably applies to the ability of polydendrocytes to give rise to astrocytes in the healthy brain. As was shown recently, polydendrocytes in the embryonic brain differentiate into astrocytes, but this capability is not maintained into adulthood [44].

In our study we also detected EGFP^+^ oligodendrocytes before and after ischemia; however, in comparison to the large number of other newly-derived cell types, newly generated oligodendrocytes did not comprise the major cell population that originated from polydendrocytes after ischemia. The low number of EGFP^+^ oligodendrocytes in the gray matter of the healthy brain is in agreement with previous studies describing the generation of oligodendrocytes predominantly in the white matter [10], [45]. Nonetheless, we also detected a low number of oligodendrocytes after ischemia, which accords well with recent findings [16], but is in contrast to other recently published studies [10], [15]. Importantly, we employed a more severe model of injury in which high levels of glutamate are released and which results in extensive neuroinflammation, and this might be an adverse environment for oligodendrocyte survival, which are strongly susceptible to excitotoxic death [46].

The last type of NG2^+^/EGFP^+^ cells found in uninjured and ischemic brains are the often overlooked pericytes. Since these cells express both NG2 and PDGFαR [47], they are probably involved in every fate mapping study that uses these proteins as markers of polydendrocytes. In our study pericytes were identified based on PDGFβR expression and the absence of any voltage activated currents; fourteen days after ischemia, their number almost doubled when compared to controls. Although we did not perform a detailed immunohistochemical analysis, there was an evident increase in the number of EGFP^+^ cells that displayed a pericytic morphology and lined vessels in the vicinity of the ischemic lesion. Of note, a recent study described a massive proliferation of a distinct subclass of pericytes and their participation in scar tissue formation following spinal cord injury [48]. In contrast to these findings, we did not detect any EGFP^+^ pericytes inside the ischemic core, which might be caused by our employing a different model of injury in a different CNS region. Moreover, the existence of several types of pericytes was described previously [49], [50], therefore the NG2^+^ pericytes labeled in our study could have a different role following ischemic injury. Recently, the wide differentiation potential of pericytes was demonstrated *in vitro* [51], so we cannot exclude their contribution to the generation of different cell phenotypes after ischemia.

It is probable that polydendrocytes close to the ischemic region are exposed to factors and signals that indicate that tissue homeostasis and the integrity of the blood–brain barrier has been disrupted. Which components present in the serum, or which signals released from neural and non-neural elements are responsible for their increased proliferation and/or fate decision, remains to be determined. Nevertheless, these factors are likely to activate the Notch-1 pathway, as suggested by its up-regulation in reactive glial cells after injury [52]–[54]. A similar up-regulation was described for bone morphogenetic factor in the post-injury niche, and this factor was shown to drive the differentiation of polydendrocytes into astrocytes, while simultaneously its antagonist Noggin reverses this process [55]. A very important role in controlling the proliferation and differentiation of polydendrocytes is also played by the β-catenin signaling pathway, which is strongly activated after cortical injury [56]. It seems that information about massive ischemic injury is delivered to polydendrocytes in a large part of the CNS; however, only the directly exposed subpopulation of polydendrocytes responds to this pathology not only by proliferation, but also by differentiation into another cell types.

## Supporting Information

Figure S1 **Enhanced green fluorescent protein (EGFP) is expressed also in pericytes, but not in microglia in the control dorsal cortex of NG2creBAC:ZEG mice.** A, Immunostaining for PDGFβR in the cortex showing several PDGFβR^+^/EGFP^+^ cells with morphology of pericytes. White arrows highlight several examples. B, Image showing immunostaining for microglial marker Iba-1; however, EGFP^+^ cells never express this marker. Scale bars, 50 µm.
(TIF)

Figure S2 **Proliferation and multipotency of EGFP^+^ cells after MCAo.** A, Immunostaining for PCNA in the cortex showing almost all EGFP^+^ cells expressing this proliferation marker 3 days after MCAo. White arrows highlight several examples. B, Image showing all channels from Fig. 3H and orthogonal projection of this EGFP^+^/GFAP^+^ cell 7 days after MCAo. C, Several EGFP^+^/GFAP^+^ cells proliferate 7 days after MCAo based on Ki-67 expression. D, Image showing in detail EGFP^+^ cells with strong expression of doublecortin (DCX) 7 days after MCAo. Scale bars, 50 µm.
(TIF)

Figure S3 **Immunohistochemical properties of EGFP^+^ cells after MCAo.** A, Immunostaining for Pax-6 in the cortex showing several EGFP^+^ cells expressing this transcription factor 7 days after MCAo. White arrows highlight several examples. B, Image showing a CD11b staining on EGFP^+^ cells 7 days after MCAo. The ischemic region is filled with activated microglia cells; however, no microglia expressed EGFP. C, Image showing a Iba-1 staining on EGFP+ cells 7 days after MCAo, also this marker of microglia and macrophages was not co-expressed with EGFP. D, Several EGFP^+^/DCX^+^ cells expressed apoptotic marker caspase-3 14 days after MCAo. Scale bars, 50 µm.
(TIF)
