# Supplementary figures and images for: Polydendrocytes Display Large Lineage Plasticity following Focal Cerebral Ischemia
Source: PLoS One. 2012 May 10;7(5):e36816. doi: 10.1371/journal.pone.0036816 (PMC3349640; doi:10.1371/journal.pone.0036816)

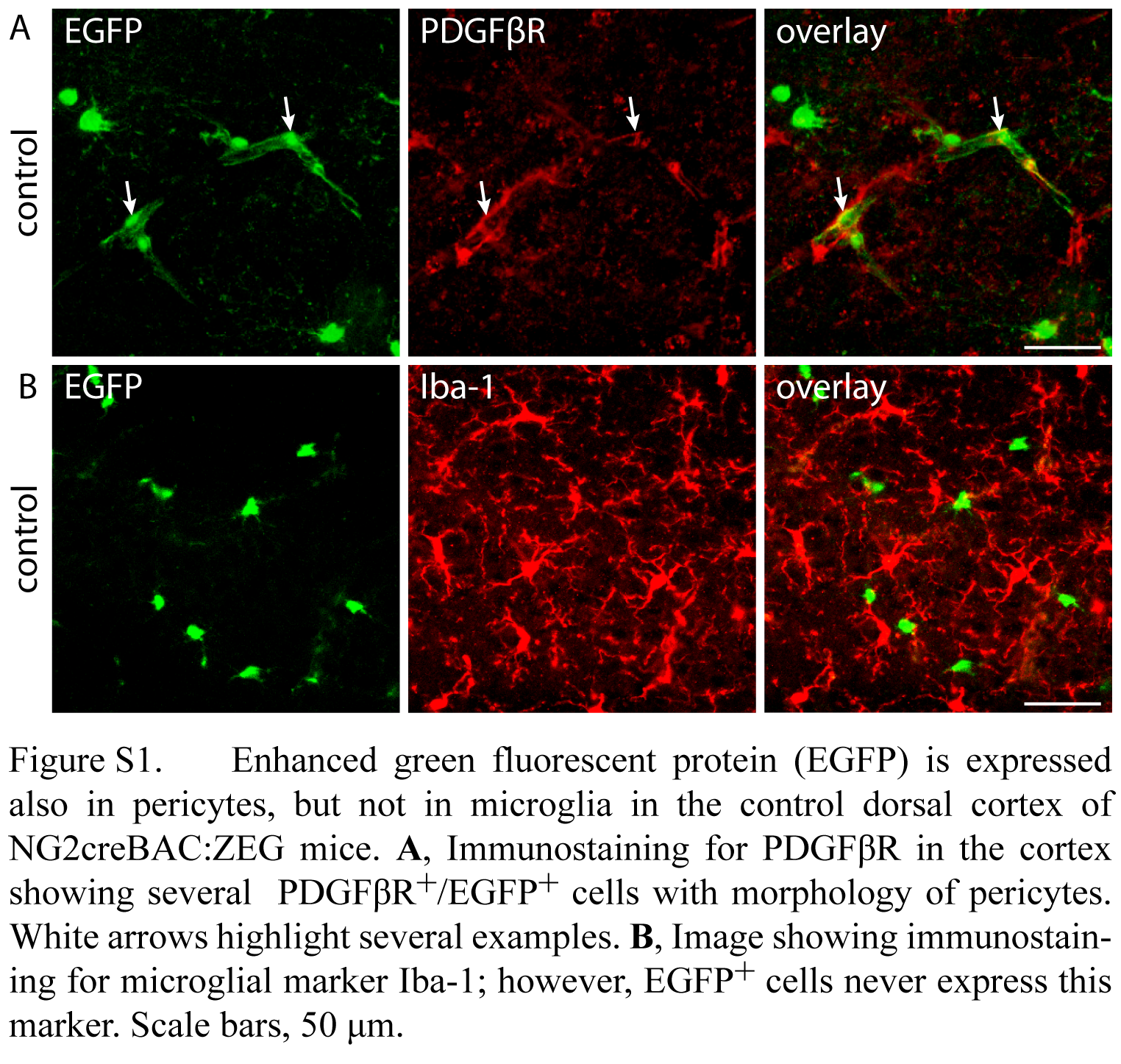

Supplement: Figure S1 — Enhanced green fluorescent protein (EGFP) is expressed also in pericytes, but not in microglia in the control dorsal cortex of NG2creBAC:ZEG mice. A, Immunostaining for PDGFβR in the cortex showing several PDGFβR+/EGFP+ cells with morphology of pericytes. White arrows highlight several examples. B, Image showing immunostaining for microglial marker Iba-1; however, EGFP+ cells never express this marker. Scale bars, 50 µm. (TIF) [file pone.0036816.s001.tif]

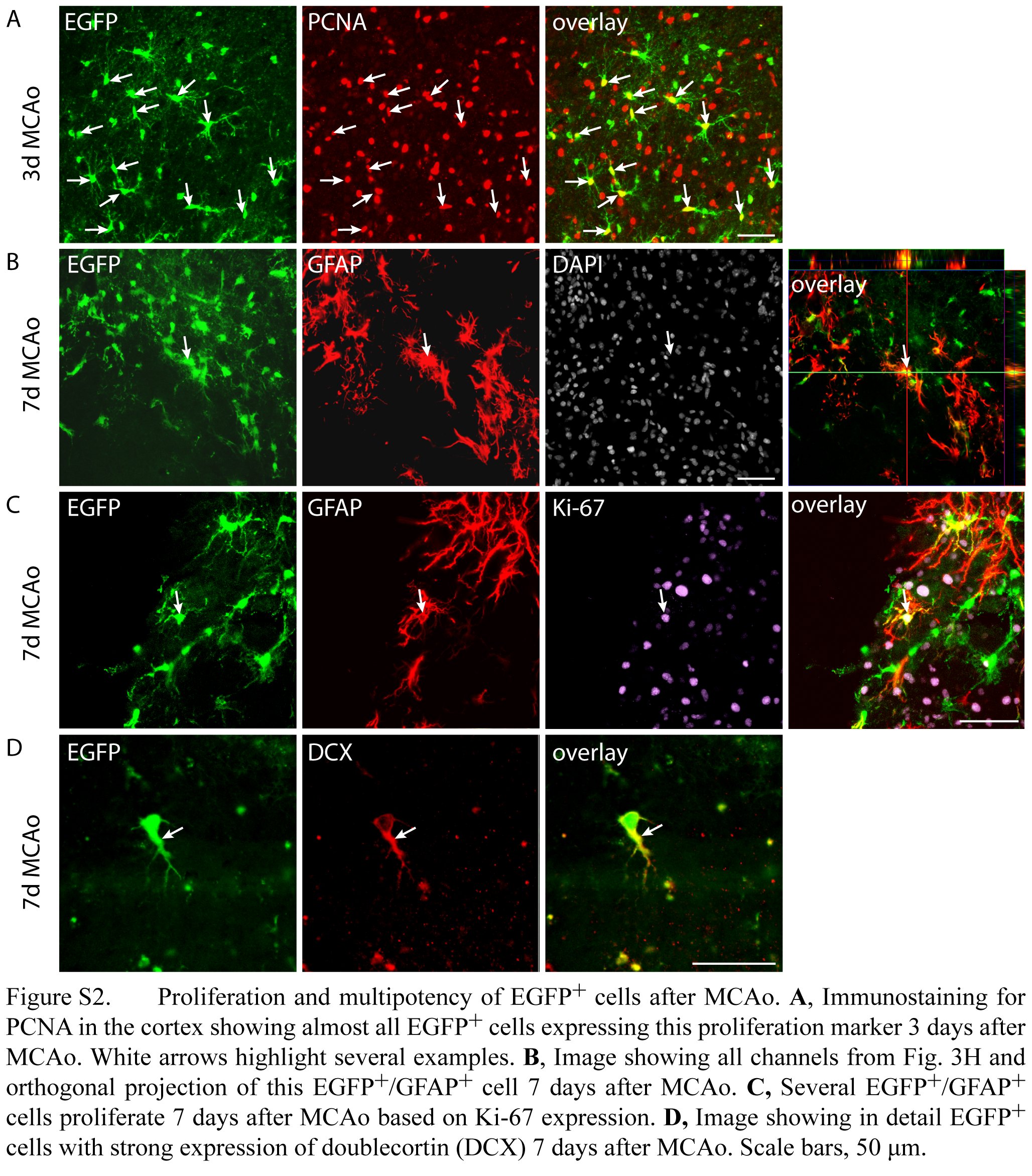

Supplement: Figure S2 — Proliferation and multipotency of EGFP+ cells after MCAo. A, Immunostaining for PCNA in the cortex showing almost all EGFP+ cells expressing this proliferation marker 3 days after MCAo. White arrows highlight several examples. B, Image showing all channels from Fig. 3H and orthogonal projection of this EGFP+/GFAP+ cell 7 days after MCAo. C, Several EGFP+/GFAP+ cells proliferate 7 days after MCAo based on Ki-67 expression. D, Image showing in detail EGFP+ cells with strong expression of doublecortin (DCX) 7 days after MCAo. Scale bars, 50 µm. (TIF) [file pone.0036816.s002.tif]

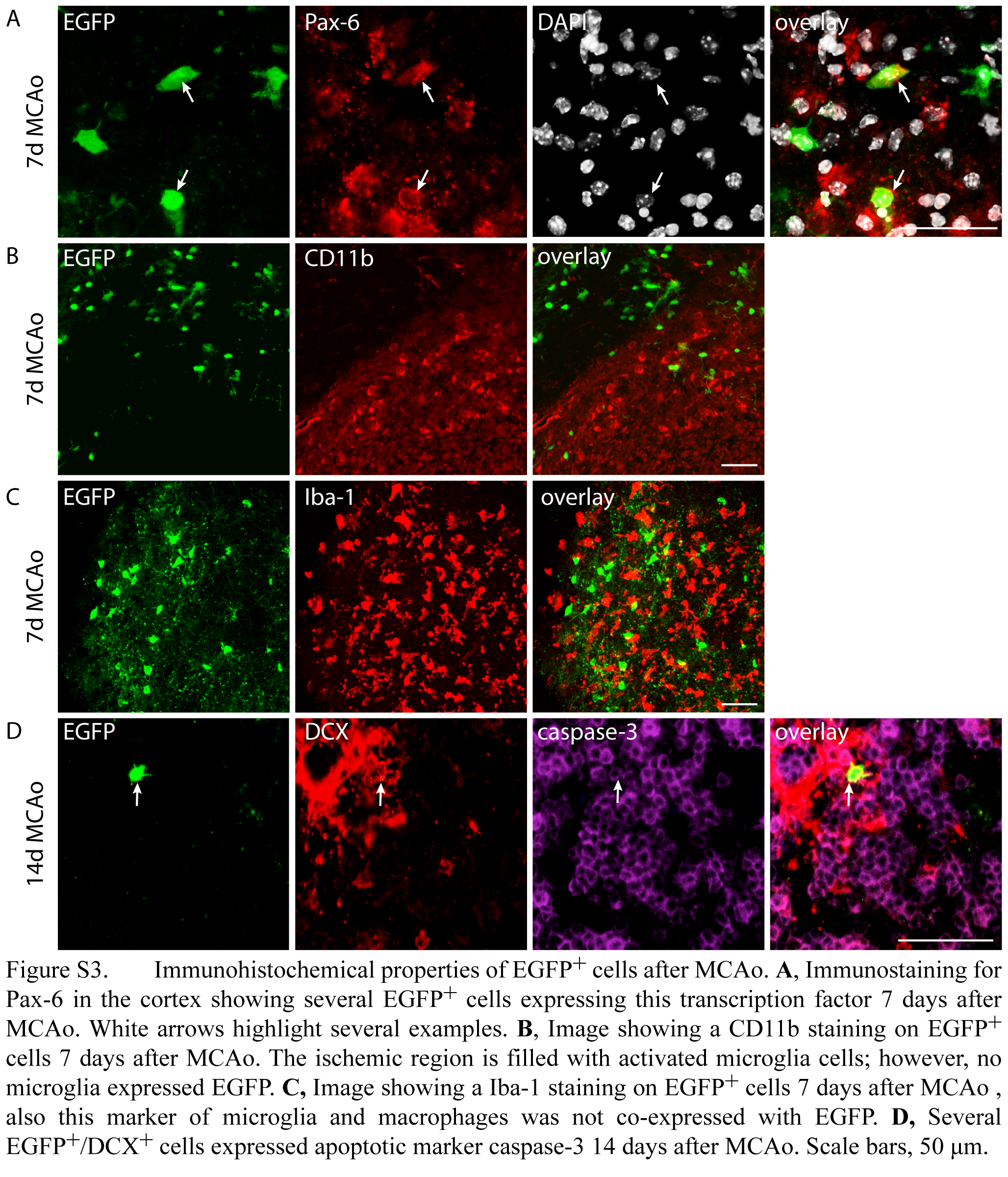

Supplement: Figure S3 — Immunohistochemical properties of EGFP+ cells after MCAo. A, Immunostaining for Pax-6 in the cortex showing several EGFP+ cells expressing this transcription factor 7 days after MCAo. White arrows highlight several examples. B, Image showing a CD11b staining on EGFP+ cells 7 days after MCAo. The ischemic region is filled with activated microglia cells; however, no microglia expressed EGFP. C, Image showing a Iba-1 staining on EGFP+ cells 7 days after MCAo, also this marker of microglia and macrophages was not co-expressed with EGFP. D, Several EGFP+/DCX+ cells expressed apoptotic marker caspase-3 14 days after MCAo. Scale bars, 50 µm. (TIF) [file pone.0036816.s003.tif]
